# Systematic review of the association between isolated musculoskeletal hypermobility and adolescent idiopathic scoliosis

**DOI:** 10.1007/s00402-022-04508-z

**Published:** 2022-07-16

**Authors:** Clare Shere, Emma M. Clark

**Affiliations:** Musculoskeletal Research Unit, University of Bristol, Southmead Hospital, Bristol, UK

**Keywords:** Musculoskeletal hypermobility, Adolescent idiopathic scoliosis, Systematic review

## Abstract

**Introduction:**

Adolescent idiopathic scoliosis (AIS) affects 1–3% of the population, but its pathogenesis remains unclear. The coexistence of musculoskeletal hypermobility and scoliosis in many inherited syndromes raises the possibility that isolated musculoskeletal hypermobility may contribute to AIS development or progression.

**Methods:**

We performed a systematic review of the evidence for a relationship between isolated musculoskeletal hypermobility and AIS. A meta-analysis was planned, but if not possible, a narrative evidence synthesis was planned.

**Results:**

Nineteen studies met eligibility criteria for inclusion. One study was excluded due to insufficient quality. Substantial heterogeneity in study design and methodology negated meta-analysis, so a narrative review was performed. Of the 18 studies included, seven suggested a positive association and eight found no association. Three reported the prevalence of musculoskeletal hypermobility in individuals with AIS. Overall, there was no convincing population-based evidence for an association between musculoskeletal hypermobility and AIS, with only two case–control studies by the same authors presenting compelling evidence for an association. Although populations at extremes of hypermobility had a high prevalence of spinal curvature, these studies were at high risk of confounding. Wide variation in methods of measuring musculoskeletal hypermobility and the challenge of assessing AIS in population-based studies hinder study comparison.

**Conclusions:**

There is a paucity of high-quality evidence examining the association between isolated musculoskeletal hypermobility and AIS. Large-scale prospective studies with adequate adjustment for potential confounding factors could clarify the relationship between musculoskeletal hypermobility and AIS to elucidate its role in the pathogenesis of AIS.

## Introduction

Scoliosis is a lateral and rotational deformity of the spine. The most common type is adolescent idiopathic scoliosis (AIS), which presents after age 10 and has a prevalence between 1 and 3% [1]. Even small curves are associated with back pain both in adolescence and later life [2], and scoliosis can have considerable psychosocial impacts, particularly on body image [3]. At extremes, scoliosis can affect respiratory function [4], and severe and progressive AIS can require extensive surgery.

The pathogenesis of AIS remains unclear, but is most likely to be multifactorial. Factors including greater height, delayed puberty and late menarche in females, and low BMI have been shown to contribute [5–7]. The gold standard for diagnosis of AIS is via antero-posterior radiography, with a measurement of the Cobb angle of the scoliotic curve over 10° diagnostic of AIS [8]. Screening methods include the Adam’s forward bend test (FBT), with scoliometer measurement of the angle of trunk rotation (ATR) increasing sensitivity and specificity, and back surface topography, which uses contours visible on digital images to detect spinal deformity.

Musculoskeletal hypermobility is common, with a wide variation in prevalence, reported between 7% and 59% in adolescents [9–11]. It is more common in females and generally reduces with age [12]. It exists on a spectrum, ranging from asymptomatic hypermobility through to hypermobility spectrum disorder, with associated symptoms including joint clicking and musculoskeletal pain [13]. The most commonly used measure of hypermobility is the Beighton score, which assesses the mobility of nine joints [14]. Traditionally, a score of ≥ 4/9 hypermobile joints signifies generalised musculoskeletal hypermobility, although this cut-off may over-represent clinically important musculoskeletal hypermobility [15].

Both musculoskeletal hypermobility and scoliosis are features of inherited syndromes including Marfan’s syndrome, osteogenesis imperfecta and certain types of Ehlers-Danlos syndrome, with recognised mutations in genes encoding connective tissues [16, 17]. This observation could point towards an underlying aetiological pathway between musculoskeletal hypermobility and idiopathic scoliosis, with excessive bending and rotation of the growing spine contributing to the pathogenesis of AIS.

Delineating the underlying multifactorial pathogenesis of AIS could pave the way to identification of those at risk of both initiation and progression, to guide which individuals need closer monitoring. Given the observation of co-existence of musculoskeletal hypermobility and scoliosis in inherited syndromes, we aimed to systematically review the literature for a relationship between isolated musculoskeletal hypermobility and AIS.

## Method

### Study selection

The search strategy was constructed to identify studies investigating the relationship between isolated musculoskeletal hypermobility (not as part of an inherited syndrome) and AIS. This was applied to the databases MEDLINE, EMBASE, CINAHL, AHMED and PsychInfo, from inception to September 2021. Forward and backward searches (Google Scholar cited reference search and screening reference lists) were performed on eligible studies.

Search terms for musculoskeletal hypermobility used the corresponding subject heading for each database, and the text words hypermob* or laxity or flexibil* or GJH or GJL or JHS or HSD or "hypermobile Ehlers-Danlos syndrome" or hEDS or EDS-HT or "Ehlers-Danlos type III" or "EDS type III" or "Ehlers-Danlos syndrome type 3" or "EDS type 3". Hypermobile Ehlers-Danlos syndrome was included due to its close clinical overlap with hypermobility spectrum disorder and the absence of a known specific genetic association, making it arguably part of the spectrum of musculoskeletal hypermobility [13]. Search terms for AIS used ‘scoliosis’ as subject heading and text word.

Studies were eligible for inclusion if they assessed the relationship between musculoskeletal hypermobility using any clinical measure of generalised hypermobility, and AIS measured via X-ray or screening methods. Studies assessing musculoskeletal hypermobility or scoliosis as part of an inherited syndrome were excluded. Studies assessing the mobility of a single area of the musculoskeletal system were excluded, as this may only represent localised musculoskeletal hypermobility, and there is often poor correlation between hypermobility in a single area and a diagnosis on the spectrum of generalised musculoskeletal hypermobility [18, 19]. Case reports, case series and conference abstracts were excluded. There was no limit on year of publication or language. The review was registered on PROSPERO on 12/8/21, registration number CRD42021206072.

Records retrieved were screened by title and abstract by CS using Endnote. Full-text articles identified were screened independently by CS and EC based on eligibility criteria. Any discrepancies were resolved through discussion. Characteristics of eligible studies were collected using a standardised Excel spreadsheet (study year, population, sample size, methods of diagnosis of musculoskeletal hypermobility and AIS, curve types, Cobb angles and outcome).

### Analysis

Eligible studies were assessed for quality using the Newcastle–Ottawa Scale (NOS) [20] by CS, with uncertainties resolved through discussion with EC. Based on standard classifications of this scale, studies were classified as at high risk of bias if the score was < 5. Those with a score of < 3 were excluded from further analysis. Studies were then assessed for heterogeneity in study design and methods of identification and measurement of hypermobility and AIS, to determine whether meta-analysis was possible. Otherwise, a narrative evidence synthesis was planned. Weighting of studies within any narrative synthesis was performed based on the hierarchy of evidence (study design) and NOS score.

## Results

**Fig. 1:**
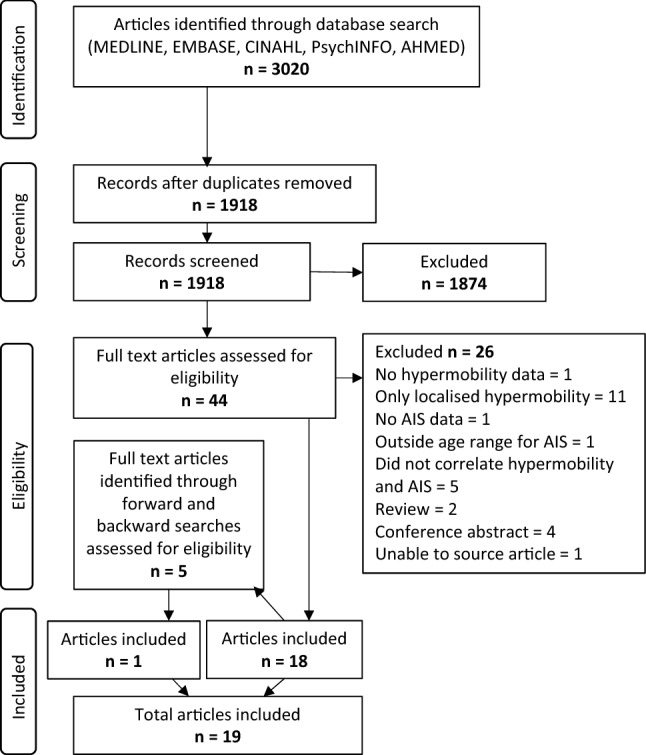
PRISMA diagram for selection of eligible studies

The PRISMA diagram in Fig. 1 shows articles retrieved and screened.

Nineteen studies fulfilled the eligibility criteria (see Table 1); 26 were excluded after full-text review because they either did not include extractable data on hypermobility or AIS, were outside the age range, were conference abstracts or non-relevant reviews or did not examine the association between hypermobility and AIS.

**Table 1:** Summary of eligible studies arranged by study design

	Population	Case definition	Control definition	Inclusions	Exclusions	Sample size	Age (years)	Measure of hypermobility	Measure of scoliosis	Outcome	Curve types	Cobb angle
*Case–control studies*												
Weber [28]	Germany. Scoliosis clinic	Scoliosis patients undergoing Milwaukee brace treatment	Healthy individuals in the same age range			74 cases vs control group not reported	Range 7–18	Carter and Wilkinson criteria + nine more tests: tongue-nose test, thumb sign, swan-neck phenomenon, heel-belly button test, ability to spread hip joints (distance between thighs to leg length ratio), knee joint anterior draw test, presence of pes planus, spine lateral and forward flexion	Diagnosed in scoliosis clinic	Prevalence using cut-off of ≥ 3/14 for hypermobility was 62% in scoliosis group vs 77% in control group. Hypermobility scores roughly equal between groups. Increased levels of thumb hypermobility, ankle hyperextension, reduced levels of knee hyperextension and hip abduction in scoliosis group compared with control group. Spine lateral flexion increased towards the convex side with increasing duration of brace treatment. No association between severity of scoliosis or age of onset and hypermobility	Not reported	Not reported
Mattson [19]	Sweden. Idiopathic scoliosis clinic and a school	Female untreated mild idiopathic scoliosis	Females with presumed structurally normal spines			116 (51 cases vs 65 control)	Range 10–16 (mean 13 scoliosis vs 13.5 control)	7 measures: index extension, wrist bend, elbow hyperextension, knee hyperextension (goniometers), spine forward flexion (difference in C7-S1 distance ratio), trunk lateral bend (fingertip to floor). Side of body selected at random	Cobb angle from clinical records	Females with idiopathic scoliosis were no more flexible, in some tests less flexible (index finger extension, spine forward flexion and right lateral flexion). Neither patients with severe or progressive curves had different flexibilities. Poor correlation of relative flexibility between joints	6 single thoracic, 12 single thoracolumbar, 6 single lumbar	Mean 16.6°
Veldhuizen [33]	Netherlands	Female untreated mild idiopathic scoliosis	Females with presumed structurally normal spines			20 (10 cases vs 10 control)	Range 10–16 (mean 13.2 AIS group vs 12.8 control)	7 measures as Mattson (except spine flexion C5-S1 distance) plus new measure of bending stiffness of trunk—apply load to trunk and measure rotation at shoulder region while supine	Cobb angle via X-ray	Poor correlation of the 7 measures of flexibility, wide ranges of each measure. Based on means of each measure, AIS group tended towards being less flexible. No significant difference in trunk bending stiffness	Not reported	Mean 22.6°
Fuller [34]	USA schoolgirls	Females with idiopathic scoliosis	Normal spines		No 'handicapping condition'	96 (48 cases vs 48 control)	Mean 12.3 AIS vs 12.8 control	4 flexibility tests: Leighton flexometer for range of movement of (1) shoulder flexion + extension (2) shoulder abduction + adduction (3) knee flexion + extension (4) trunk lateral flexion + rotation	AIS diagnosed by their physician	No significant association between the 4 flexibility measures. AIS group had a trend towards reduced mean trunk flexibility to the dominant side (AIS group 268 vs control 280, *p* = 0.068)	Not reported	Not reported
Fernandez-Bermejo [37]	Madrid, Spain. 121 from scoliosis clinic (74 from a screening program for hypermobility and scoliosis of 600 patients)	AIS or musculoskeletal hypermobility	Not defined		Neurological disorder	AIS group 52 (77% female)	AIS group range 13–19 (mean 14.9)	Beighton ≥ 5/9	Cobb angle > 10° via X-ray	12/52 in AIS group were hypermobile (23%)	9 single thoracic, 14 thoracolumbar, 13 thoracic and lumbar double curve, 12 lumbar, 4 other	Range 10–35°. 65% had curves 10–19°, 35% had curves 20–35°
Tanchev [23]	Bulgaria	Female rhythmic gymnasts	4800 11-15 yr old Bulgarian females from a screening program performed by the same team	Practiced rhythmic gymnastics for > 5yrs	Family history of spinal deformity, diseases or congenital abnormalities resulting in secondary scoliosis	100 cases vs 4800 control	Rhythmic gymnasts mean 12.44 (range 10–16) vs Bulgarian females mean 13 (range 11–15)	Carter and Wilkinson: > 3 of (both upper and lower limb): (1) passive apposition of the thumb to the flexor aspect of the forearm, (2) passive hyperextension of the fingers so that they lie parallel with the extensor aspect of the forearm, (3) ability to hyperextend the elbow more than 10°, (4) ability to hyperextend the knee more than 10°, (5) excess range of passive dorsiflexion of the ankle and eversion of the foot	FBT—if positive, X-ray	FBT positive in 16/100— > XR—12 had scoliosis with Cobb > 10°. Prevalence of AIS was 12% in rhythmic gymnasts vs 1.1% in adolescent Bulgarian girls. 100% of rhythmic gymnasts had musculoskeletal hypermobility vs 5% of control group	58% thoracolumbar, 42% lumbar. 67% right convexity	Mean 16° (range 10–30°)
Czaprowski [21]	Poland	Radiological AIS	ATR < 5° via Bunnel scoliometer	Age 9–18	Systemic diseases related to hypermobility (Ehlers-Danlos, Downs, Marfan, Larsen)	128 (70 cases vs 58 control (71.9% female))	Mean 13.2 AIS group vs 12.6 control	Beighton score and five-part questionnaire by Hakin and Graham. Hypermobility = Beighton ≥ 4/9 + questionnaire ≥ 2/5	Cobb angle > 10° via X-ray in AIS group	AIS group had higher prevalence of hypermobility (51.4%vs 19%, *p* < 0.001), for Males and females. Hypermobility more common in those with single curves vs double curves (64.7% vs 39%, *p* = 0.03). No significant differences in hypermobility between mild or moderate scoliosis, no associations between hypermobility and Cobb angle, Beighton score, treatment type (physio or physio + brace) or number of vertebrae in curve	34 single curve thoracic, 36 double curve thoracic and lumbar	Range 10–34°
Kobesova [27]	Rehabilitation and sports medicine clinic, Czech Republic	Radiologically confirmed idiopathic scoliosis	No scoliosis		Pain during measurement, neurological or orthopaedic diseases other than AIS, surgery for AIS	22 (11 cases vs 11 control (73% female))	AIS range 14–66 (mean 31.54) vs control range 14–64 (mean 34.45)	10 tests: head rotation, scarf test (reach arm behind opposite side of neck), touching hands behind back, crossing hands behind neck, elbow extension, clasped hands test (wrist extension), clasped fingers test, forward bending test, side bending test, heels sitting test. Each test graded hypomobile/normal, slight hypermobility or hypermobile	Cobb angle > 10° via X-ray in AIS group	AIS group more mobile in all tests but only 2/10 tests significantly different (head rotation and forward bending test). Difference in total scores for all tests 255 AIS vs 174 control (*p* = 0.051)	Not reported	Not reported
Czaprowski [22]	Poland. Controls from 8 schools	Females with radiological AIS	Females with ATR < 5° via Bunnell scoliometer	Age 9–18 years	Systemic diseases related to hypermobility (Ehlers-Danlos, Downs, Marfan, Larsen), musculoskeletal pain throughout previous 6 months	356 (155 cases vs 201 control)	AIS mean 13.8 vs control mean 14	Beighton score ≥ 5/9	Cobb angle > 10° via X-ray in AIS group	Hypermobility more common in AIS group (36 (23.2%) vs 27 (13.4%), *p* = 0.02). Significant negative association between points in Beighton score and age, not Cobb angle. No differences in hypermobility by curve severity, number of vertebrae in curve or curve type	44 single thoracic, 35 single lumbar, 76 double curve	Range 11–65° (mean 28.2°). 74 mild (11–24°), 57 moderate (25–40°), 24 severe (> 40°)

	Population	Inclusions	Exclusions	Sample size	Age (years)	Measure of hypermobility	Measure of scoliosis	Outcome	Curve types	Cobb angle
*Cross-sectional studies in the general population*										
Erkula [25]	Turkey. School children			1273 (47% female)	Range 8–15 (mean 10.4)	Beighton score ≥ 7/10 (2 points for trunk forward flexion)	ATR using Bunnell scoliometer. If ≥ 7°, X-ray. Also scapular asymmetry	30/1273 (2.3%) had ATR ≥ 7°, then 10/30 had AIS via XR (Cobb 11–18°)—prevalence 0.78%. 41/1273 (3.2%) had Beighton score ≥ 7. Hypermobile group had significantly higher ATRs (2.31 ± 3.21° vs 1.29 ± 2.02°, *p* = 0.039). Beighton scores higher in the AIS group (*p* = 0.023)	7 thoracic, 2 thoraco-lumbar, 1 lumbar	Range 11–18°
Farro-Uceda [29]	All students in 5th year of a private secondary school, Lima, Peru	Males and females from 5th year	Previous central or peripheral vestibular problem, head trauma, neurological, osteo-articular or muscular problems	247 (44.1% female)	Range 14–17 (mean 15.2)	Beighton score ≥ 5/9 = generalised hypermobility, Beighton score 1–4 = localised hypermobility, Beighton score 0 = not hypermobile	Cobb angle > 10° via X-ray	Generalised hypermobility prevalence 9.7%. Scoliosis prevalence 17.8%. 65.6% had curves < 10°. No significant difference in prevalence of scoliosis or smaller curves between hypermobile group (Beighton score 1–9) vs non-hypermobile group (Beighton score 0) (14.1% had scoliosis in hypermobile group vs 20% in non-hypermobile group, 69.6% had curves < 10° in hypermobile group vs 63.2% in non-hypermobile group)	Not reported	Range 11–30°
Dolphens [30]	64 schools in Flanders, Belgium	Males in year 1 of secondary education, females in year 5 of primary education	Neurological, rheumatic, metabolic or endocrine diseases, major congenital abnormalities, skeletal disorders, connective tissue disorders, previous spinal fracture or spinal surgery, apparent severe spinal asymmetry, radiographically confirmed scoliosis	1196 (47% female)	Males mean 12.6, females mean 10.6	Beighton score ≥ 4/9	Back surface topography	Coronal plane trunk asymmetry in 21%, no sex difference. No association between hypermobility and trunk asymmetry (OR for trunk asymmetry if hypermobile in males 0.68 (95% CI 0.36,1.32) *p* = 0.255, in females 0.89 (95% CI 0.50,1.57) *p* = 0.442)—adjusted for trunk lean angle, thoracic kyphosis, number of vertebrae in the declive thoracolumbar segment, sacral inclination, BMI	18.4% thoracic curves, 12% thoracolumbar curves, 69.6% double thoracic and thoracolumbar curves	N/A
Bozkurt [31]	8 secondary schools, Ankara, Turkey			822 (49.8% female)	Mean 12.2 (range 10–15)	Beighton score ≥ 4/9	Adam's FBT and ATR ≥ 5° with Bunnell scoliometer—X-ray if positive. Positive if Cobb angle ≥ 10°	Prevalence of generalised hypermobility 18.4%, no significant sex difference. Prevalence of AIS 43/822 (5.2%), of which 10 hypermobile, 33 normal. No association between hypermobility and AIS (*p* = 0.71)	16 single left thoracolumbar, 12 single left lumbar	All mild AIS, except 1 severe
Pratelli [26]	11,820 school students in Florence, Italy			11,820 (49% female)	Range 9–18	Beighton score	ATR ≥ 5° via Bunnell scoliometer or hump height ≥ 5 mm	Prevalence of clinical spinal curvature 14.05%. Prevalence of ATR ≥ 5° or hump height ≥ 5 mm was 2.03% (clinical evidence of scoliosis). Mean Beighton score in those with no clinical evidence of spinal curvature was 1.96 vs 2.41 in those with clinical scoliosis	Left lumbar most common (31%) followed by right thoracolumbar (16%)	N/A

	Population	Inclusions	Exclusions	Sample size	Age (years)	Measure of hypermobility	Measure of scoliosis	Outcome	Curve types	Cobb angle
*Cross-sectional studies in specific populations*										
Longworth [32]	Dance school, Australia	Age 9–16. Dancers: at least 3 years dance experience, at least 4 h training per week, can provide an age-matched non-dancer as control	Control group: involvement in dance, gymnastics, calisthenics	60 (30 female dancers vs 30 female controls)	Mean 12 ± 2.6 dancers vs 12 ± 2.5 control	Beighton score ≥ 4/9	ATR via Bunnell scoliometer (positive if visible hump or ATR > 5° on mean of 3 readings)	Hypermobility more common in dancers (21/30 vs 1/30, *p* = 0.04). AIS more common in dancers (30% of dancers vs 3.33% of controls, OR for having scoliosis in dancers = 12.43, *p* = 0.006). In dancer group, no significant associations between AIS and Beighton score (OR 1.23 (CI 0.86–1.75), *p* = 0.25)), age of menarche or BMI, trend towards positive association with hours of dance practice	N/A	N/A
Steinberg [24]	Female dancers from three schools with specialised dance programme, Israel	Fully active in dance classes past 3 months	Previous knee surgery, absence from class due to pain/injury for > 3 days	132 females	Range 12–14	Beighton score ≥ 5/9	Adam's FBT—if positive, Magee's sykline view test	Prevalence of musculoskeletal hypermobility 40.9% (54/132), scoliosis 28.8% (38/132), both hypermobility and scoliosis 25.8% (34/132). 4/132 (3%) had scoliosis without hypermobility. 20/132 (15.2%) were hypermobile without scoliosis. Scoliosis + hypermobility group vs no scoliosis + no hypermobility group—reduced anterior balance, reduced proprioception, weaker knee extensors and flexors	N/A	N/A

	Cohort description	Inclusions	Exclusions	Sample size	Age (years)	Measure of hypermobility	Measure of scoliosis	Outcome	Curve types	Cobb angle
*Cohort studies*										
Adib [38]	Mixed prospective/retrospective design. Prospective group: referrals to specialist hypermobility clinic at Great Ormond Street Hospital, London. Retrospective group: screening of electronic notes from other paediatric rheumatology clinics or wards	< 18yrs old, joint hypermobility diagnosed by consultant rheumatologist, adverse symptoms related to joint hypermobility	Pathological condition with joint hypermobility as a known feature, co-existent rheumatological illness which could account for some musculoskeletal symptoms	125 (54% female)	Range 3–17 (median 12)	Beighton score, no cut off. Full musculoskeletal clinical examination	Not reported	Beighton score range 2–9/9, skewed towards higher scores. 94% scored ≥ 4/9. Scoliosis reported in 10/118 (9%)	Not reported	Not reported
Hasankhani [35]	Prospective design. Surgically treated individuals with AIS, Iran		< 2 years follow up	72 (8 lost to follow up excluded, 3 with underlying syrinx excluded) (75% female)	Range 12–22 (mean 16.4)	Beighton score ≥ 5/9	Cobb angle > 10° via X-ray, some had MRI	Prevalence of hypermobility 66.6%. Hypermobile group had smaller pre-operative curves (66.73° vs 71.17°, *p* = 0.52), greater pre-operative spinal flexibility (41.4% ± 16.5 vs 31.2% ± 13.3, *p* = 0.01) and smaller post-operative curves (17.84° ± 4.48 vs 30.52° ± 9.15, *p* = 0.013) with greater post-operative curve correction (73.3% vs 58.1%, *p* = 0.001). Authors suggest hypermobility is a good prognostic indicator for surgery so could consider a less aggressive surgical approach	Not reported	Mean 71.17° in non-hypermobile group, 66.73° in hypermobile group
Haller [36]	Females with juvenile or adolescent idiopathic scoliosis from two orthopaedic surgery clinics, USA with recruitment over 10 years	Cobb angle ≥ 10°, aged 12–25 years	Males, developmental delay, multiple congenital anomalies or known underlying genetic disorders	570 females	16.48 (SD 2.77)	Beighton score ≥ 4/9	Cobb angle > 10° via X-ray	141/570 (25%) were hypermobile. Beighton scores skewed (more had smaller scores). Mean Beighton score smaller in operated vs non-operated group (1.85 ± 2.02 vs 2.39 ± 1.96, *p* = 0.0001). Weak negative correlation between Beighton score and Cobb angle, attenuated but remained after adjustment for age (rs *p* = −0.103, *p* = 0.014). Hypermobility was not a predictor of surgery. Being unable to touch palms to floor resulted in 2.5 × increased risk of surgery after adjustment for age (OR 2.5 CI 1.37, 4.6, *p* = 0.003)	Not reported	Mean 44.65° (range 15–105°). Operated group mean 61.8°, non-operated group mean 33.05°

*FBT* Adam’s forward bend test, *ATR* angle of trunk rotation, *AIS* adolescent idiopathic scoliosis

Of the fifteen studies which used a control comparator, or directly correlated musculoskeletal hypermobility and AIS, seven suggested a positive association [21–27], and eight found no association [28–32], or trends towards a negative association [19, 33, 34].

Of the three studies which reported the prevalence of hypermobility individuals with AIS, one found a high prevalence [35], and two found a prevalence within the reported range for that geographical area [36, 37]. One low-quality study reported the prevalence of scoliosis in a group with joint hypermobility syndrome [38].

### Assessment of quality

NOS scores are presented in Table 2. Scores ranged from two to seven, with nine out of the 19 studies scoring < 5 and therefore at high risk of bias. A common aspect which could introduce bias was a lack of adjustment for factors which are recognised as associated with both musculoskeletal hypermobility and AIS, including age, height, BMI and pubertal stage. Only five studies attempted some participant matching or adjustment for confounders [30, 32, 34, 36, 37]. In the case–control studies, only three out of nine attempted to exclude AIS in controls [21, 22, 33].

**Table 2:** Newcastle–Ottawa Scores for eligible studies

	Selection	Comparability	Outcome	Total
Weber [28]	*		***	4*
Mattson [19]	**		***	5*
Veldhuizen [33]	*		***	4*
Fuller[34]	*	*	***	5*
Fernandez-Bermejo [37]	***	*	***	7*
Tanchev [23}	**		***	5*
Adib [38]	**			2*
Erkula [25]	**		**	4*
Czaprowski [21]	**		***	5*
Hasankhani [35]	*		**	3*
Kobesova [27]	*		***	4*
Czaprowski [22]	****		***	7*
Longworth [32]	*	*	***	5*
Farro-Uceda [29]	**		**	4*
Dolphens [30]	***	*	**	6*
Haller [36]	**	*	**	5*
Bozkurt [31]	**		**	4*
Pratelli [26]	***		**	5*
Steinberg [24]	*		**	3*

The study scoring lowest on the NOS (2*) was a descriptive study, characterising features of a cohort with joint hypermobility syndrome referred to a tertiary centre in London, UK [38]. The method for determining scoliosis was not defined, and this was a highly selected population. No further evaluation of this study was undertaken, leaving 18 studies.

### Assessment of heterogeneity

To assess appropriateness of undertaking meta-analysis, a comprehensive review of study design and methods of identification and measurement of hypermobility and AIS was undertaken.

#### Study design

Of the 18 studies assessed, nine were case–control [19, 21–23, 27, 28, 33, 34, 37], seven were cross-sectional (five of which were conducted in the general population [25, 26, 29–31], and two in dancers and rhythmic gymnasts [24, 32]) and two were cohort studies in individuals with AIS [35, 36].

The studies comprised a total of 17,156 individuals; 15,559 were recruited from the general population (mainly represented by one cross-sectional study of 11,820 individuals [26]), 1,305 were recruited from hospital-based clinics, and 292 individuals were from highly selected populations of dancers and rhythmic gymnasts.

#### Diagnosis of AIS

The cohort studies in AIS patients [35, 36] and all of the case–control studies used X-ray to define cases, but only three case–control studies attempted to exclude AIS in controls, using X-rays done for other medical reasons [33], or ATR measurement [21, 22].

Of the cross-sectional studies, three used FBT with ATR measurement followed by X-ray if deemed positive [23, 25, 31], three studies used FBT with ATR measurement [26, 32], and one study used visual assessment only (FBT and Magee’s skyline view assessing visible humps or asymmetry) [24]. One cross-sectional study performed spinal X-rays on all participants, raising ethical questions [29].

#### Diagnosis of musculoskeletal hypermobility

There was wide variation in measures used to diagnose musculoskeletal hypermobility.

Twelve studies used the Beighton score [21, 22, 24, 25, 29–32, 35–37]. Five used the traditional cut-off score of 4/9 [21, 30–32, 36], and five used a cut-off of 5/9 [22, 24, 29, 35, 37]. The rationale for using a higher cut-off was justified only by Czaprowski et al. [22] as the sample was comprised of females, who have higher rates of hypermobility. Erkula et al. used a cut-off of 7, but attributed a score of 2 for trunk forward flexion, making the total possible score 10 [25]. Pratelli et al. compared mean Beighton scores rather than applying a cut-off [26].

The remaining studies assessed the mobilities of joints similar to the Beighton score, including Carter and Wilkinson criteria [23, 28], on which the Beighton score was based [39]. Four studies compared a variety of individual tests rather than defining individuals as hypermobile (see Table 1) [19, 27, 33, 34], which may reflect only localised musculoskeletal hypermobility, particularly as two studies found poor correlation between tests within the same individual [19, 33].

### Meta-analysis

Due to substantial heterogeneity in study design and methods for identification and measurement of hypermobility and scoliosis, it was not possible to undertake a meta-analysis. Therefore, a narrative synthesis was performed.

### Narrative synthesis

#### Case–control studies

Two high-quality case–control studies found a positive association between musculoskeletal hypermobility and AIS [21, 22], five lower-quality case–control studies found no association [19, 28, 33, 34], and one reported the prevalence of hypermobility as in the reported range for the area among individuals with AIS [10, 37, 40].

The highest quality case–control study was performed by Czaprowski et al., who conducted two similar studies. In the first, they found a higher prevalence of musculoskeletal hypermobility in male and female AIS patients compared to controls (51.4% vs 19.0%, *p* = 0.00015) [21]. The second study was larger, and only included females [22]. Using a higher cut-off for hypermobility (Beighton score ≥ 5/9), justified by its greater prevalence in females, they also found higher prevalence of hypermobility in AIS patients compared to controls (23.2% vs 13.4%, *p* = 0.02). Neither study found significant correlation between hypermobility and severity of AIS. The strengths of these studies were confirming similar baseline characteristics in cases and controls, robust inclusion and exclusion criteria, and a reasonable attempt to exclude AIS in controls using ATR measurements.

Four case–control studies, two of which were small (*n* = 20 [33] and *n* = 22 [27]), compared a variety of individual tests for musculoskeletal hypermobility, and in general found no differences in hypermobility between those with AIS and controls [19, 27, 33, 34]. Weber found no difference between groups using a total hypermobility score [28]. However, the low threshold is used to define musculoskeletal hypermobility (> 3/14 positive tests), and the inclusion of so many tests impacts the study quality.

#### Cross-sectional studies carried out in the general population

Of five cross-sectional studies carried out in schoolchildren, three found no association [29–31], and two found a positive association between musculoskeletal hypermobility and AIS [25, 26].

The highest quality cross-sectional study found no association between musculoskeletal hypermobility and suspected early spinal curves (measured using back surface topography) with adjustment for posture and BMI (in males OR 0.68 (95% CI 0.3–1.32, *p* = 0.255), in females OR 0.89 (95% CI 0.50–1.57, *p* = 0.442)) [30]. The study was designed to sample children just prior to the pubertal growth spurt, a high-risk period for AIS development [41]. Those with known or clearly visible scoliosis were excluded, so we can only conclude that hypermobility was not associated with early small spinal curves in this population.

In contrast, two studies pointed towards a positive association. A large Italian study (*n* = 11,820) found 2.03% of adolescents had clinical scoliosis (ATR ≥ 5° or hump size ≥ 5 mm) [26]. This group had a higher mean Beighton score compared to those without any clinical spinal curvature (2.41/9 vs 1.96/9). However, these scores are too low to represent generalised musculoskeletal hypermobility, and individuals with inherited syndromes were not excluded, which could have artificially inflated Beighton scores in those with spinal curvature. A Turkish study also found higher Beighton scores in those with radiologically diagnosed AIS, and hypermobile individuals (defined as Beighton score > 7/10) had slightly higher ATR measurements (mean 2.31° vs 1.29°, *p* = 0.039) [25].

Two studies smaller studies (*n* = 822 and *n* = 247), both with relatively high prevalence of radiologically diagnosed AIS, (5.2% and 17.8%) found no association with musculoskeletal hypermobility [29, 31].

#### Cohort studies in individuals with AIS

Of the two cohort studies in AIS patients, one found a high prevalence of hypermobility (66.6%) [35], and the other found a prevalence within the reported range for the area (25%) [36]. These populations were predominantly female operated patients, with more severe curves. One study found that despite similar curve severity, hypermobile individuals had better surgical outcomes in terms of percentage curve correction [35]. The second larger study (*n* = 570) found higher Beighton scores were weakly associated with lower Cobb angle (milder curves), which was attenuated but remained after adjustment for age. Being hypermobile did not predict the need for surgical intervention, although lack of trunk hypermobility conferred a 2.5 × increased risk of surgery [36].

#### Studies in highly selected populations

Three studies were performed in adolescent female rhythmic gymnasts and dancers [23, 24, 32], populations with observed high rates of both musculoskeletal hypermobility and AIS [7, 42].

As expected, there was a high prevalence of musculoskeletal hypermobility (40.9% and 100%) [23, 24]. There was also a higher prevalence of spinal curvature than age-matched controls (30% vs 3.33% using ATR measurement) [32], or females of the same age in that region (12% vs 1.1% using ATR then X-ray) [23]. In a small group of dancers (*n* = 30), there was no association between musculoskeletal hypermobility and spinal curvature (OR 1.23, 95% CI 0.86–1.75 *p* = 0.25), although the sample may have been too small to detect an association [32]. There is high risk of confounding in these studies, as rhythmic gymnasts were shorter, lighter, fewer had started menarche, and had reduced lumbar lordosis and thoracic kyphosis, all factors associated with AIS [5, 7, 43]. Equally, dancers with both musculoskeletal hypermobility and spinal curvature had weaker knee musculature, reduced proprioception and anterior balance compared with dancers without either phenotype [24].

## Discussion

The literature on the association between isolated musculoskeletal hypermobility and AIS shows varying results. Overall, there is no convincing population-based evidence for an association, although in a group of patients with mild AIS, there was some high-quality evidence for an association [22]. Potential explanations for this disparity could be selection bias or uncontrolled confounding. In selected populations where hypermobility is common, AIS is found more frequently, but again there is high risk of confounding in these studies.

Two cross-sectional studies found higher mean Beighton scores in those with clinically and radiologically diagnosed AIS [25, 26], but these scores were too low to represent a diagnosis of generalised musculoskeletal hypermobility. Hypermobile individuals had slightly higher ATR measurements, which could simply reflect excessive spinal mobility inducing functional reversible curves and therefore higher ATR measurements, which may not translate into progressive scoliotic curves [25]. Indeed, despite correlation with Cobb angle, ATR measurement overestimates the presence of a scoliotic curve in younger adolescents, which could in part be related to higher prevalence of hypermobility [44, 45]. Contrary to this theory, a high-quality cross-sectional study did not find an association between musculoskeletal hypermobility and small early spinal curves after exclusion of those with known scoliosis in an adjusted model [30], although the back surface topography method used here assesses spinal deformity in the coronal plane, as opposed to ATR, a measure of spinal rotation.

The only compelling evidence for an association between musculoskeletal hypermobility comes from two studies by the same authors [21, 22], who found higher rates of musculoskeletal hypermobility in individuals with mild AIS. However, the case–control design inherently risks selection bias, as the AIS group, recruited from a hospital-based clinic, may possess particular confounding characteristics associated with presentation to secondary care, possibly inducing or accentuating any associations.

Studies investigating dancers and rhythmic gymnasts, individuals at the extremes of hypermobility, have found higher rates of AIS [23, 24], although results were not adjusted for potential confounders which were also common in these populations (particularly low BMI and pubertal stage). The results could therefore represent confounding, or hypermobility may be one of a constellation of traits associated with AIS in these populations.

This review highlights important implications for future research into the association between musculoskeletal hypermobility and AIS. Firstly, standardisation of measurement methods would allow replication of results across populations. For musculoskeletal hypermobility, the most commonly used method for diagnosis is the Beighton score, with acceptable inter- and intra-rater reliability, and this should be used by future studies. However, consensus regarding the most appropriate cut-off scores for clinically important musculoskeletal hypermobility are needed [46]. For AIS, the gold-standard would be spinal radiographs in the entire study population, which would entail considerable exposure to ionising radiation, and would therefore be unethical in healthy individuals. The pragmatic use of screening methods to define AIS in a research setting could artificially increase the strength of any association if it overestimates the presence of scoliosis. This is particularly pertinent when investigating musculoskeletal hypermobility, as it is conceivable that excessive spinal mobility could give rise to functional spinal rotation while bending, resulting in a false positive ATR result. Newer imaging methods such as EOS, which reconstructs a 3D model of the spine [47], and techniques for measuring spinal curvature from DXA scans have been developed [48], which confer minimal radiation exposure, and could prove useful in accurately assessing spinal curvature in study populations.

Secondly, future studies must take into account the potential for confounding, particularly age, BMI and pubertal stage, in order to examine the true relationship between musculoskeletal hypermobility and AIS.

Lastly, there were no longitudinal data. It is therefore difficult to determine which curves will progress, a key factor influencing clinical management. Longitudinal data would allow analysis of changes in musculoskeletal hypermobility and curve development through adolescence, and better understanding of their temporal relationship. Determining whether musculoskeletal hypermobility impacts on curve progression could help identify at-risk individuals, and guide frequency of monitoring, or even clinical management if curves of hypermobile individuals behave differently, as hinted at by the finding of greater surgical curve correction in hypermobile individuals [35].

### Strengths and limitations

Strengths of this review are the inclusion of studies with a range of measures of generalised musculoskeletal hypermobility and scoliosis, reflecting the available literature, and our ability to include manuscripts written in English, German, Spanish and Italian. Limitations of this review include an inability to carry out a meta-analysis. However, a narrative evidence synthesis was performed, weighted towards studies of the highest quality. Common limitations in design of the eligible studies were identified.

## Conclusions

Although there are suggestions of an association between musculoskeletal hypermobility and AIS, there is a paucity of high-quality evidence. Greater understanding of the role of musculoskeletal hypermobility in the pathogenesis of AIS could help to identify factors involved in its initiation and progression and could lead to development of clinical tools to identify individuals most at-risk, to allow more tailored clinical management.

As highlighted by this review, further large-scale prospective studies are required with standardised measures of hypermobility and adequate consideration of potential confounding factors, to clarify the true role of isolated musculoskeletal hypermobility in AIS.
